# Supplementary material for: Association between Changes in Protein Intake and Risk of Cognitive Impairment: A Prospective Cohort Study
Source: Nutrients. 2022 Dec 20;15(1):2. doi: 10.3390/nu15010002 (PMC9824869; doi:10.3390/nu15010002)
Supplement: Supplementary file 1 [file nutrients-15-00002-s001.zip › nutrients-2076189-supplementary.pdf]

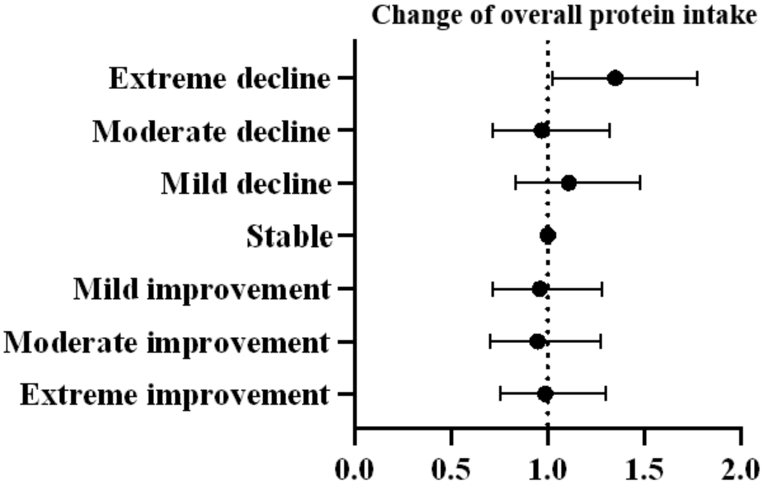

Figure S1. Association between the change patterns of overall protein intake and cognitive impairment based on restricted cubic spline model.

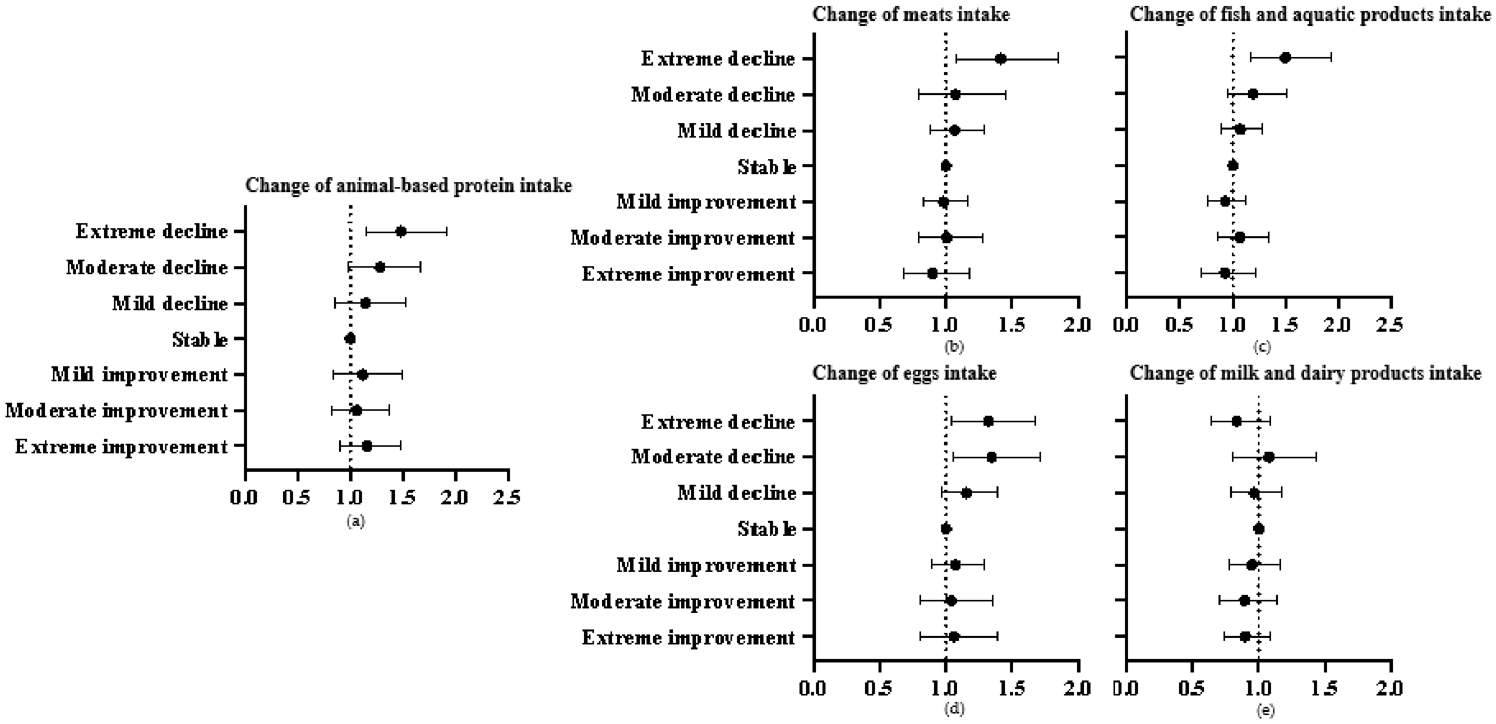

Figure S2. Association between the change patterns of protein intake and cognitive impairment based on restricted cubic spline model after adjusting for age (continuous), gender, residence, years of schooling, marital status, economic status, living pattern, tobacco smoking, alcohol drinking, regular exercise, ADL, IADL, BMI, chronic disease (hypertension, diabetes, heart disease, stroke or CVD, cataract, digestive system disease, arthritis, Parkinson's disease). (**a**) change in animal-based protein intake; (**b**) change in meats intake; (**c**) change in fish and aquatic products intake; (**d**) change in eggs intake; (**e**) change in milk and dairy products intake.

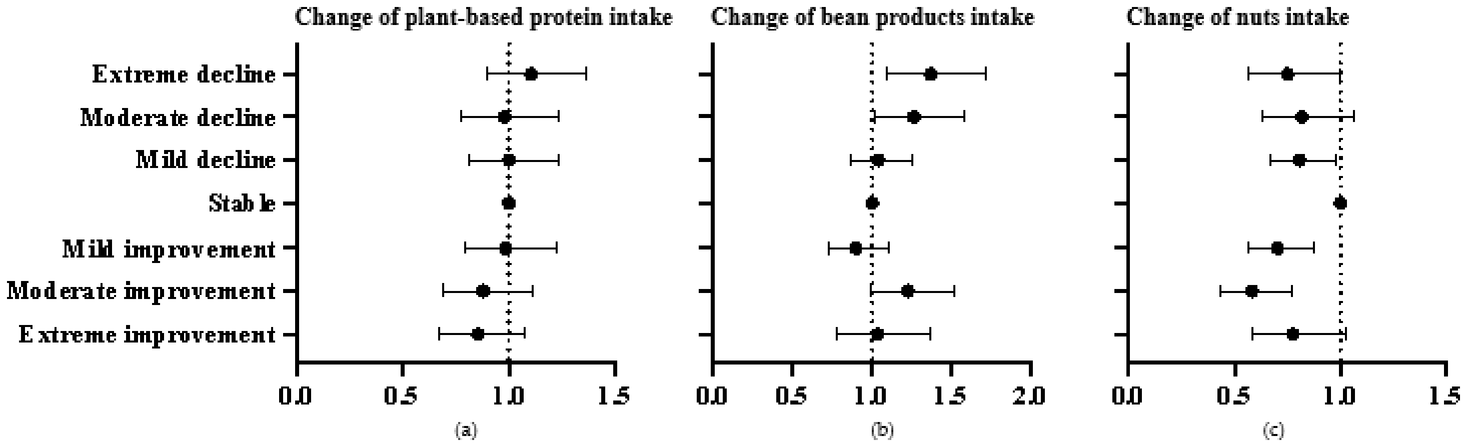

Figure S3. Association between the change patterns of protein intake and cognitive impairment based on restricted cubic spline model after adjusted for age (continuous), gender, residence, years of schooling, marital status, economic status, living pattern, tobacco smoking, alcohol drinking, regular exercise, ADL, IADL, BMI, chronic disease (hypertension, diabetes, heart disease, stroke or CVD, cataract, digestive system disease, arthritis, Parkinson's disease). (**a**) change in plant-based protein intake; (**b**) change in bean products intake; (**c**) change in nuts intake.

Table S1. The associations between changes in variables and cognitive impairment

Variables	HR (95% CI)	P value
**Age in years**	1.06 (1.05-1.07)	<0.001***
**Sex**		
Female	[1]	
Male	0.88 (0.72-1.07)	0.192
**Years of schooling**	0.93 (0.90-0.96)	<0.001***
**Location of residence**		
Rural	[1]	
Urban	0.98 (0.84-1.15)	0.828
**Change in marital status**		
Keep married	[1]	
Keep divorced/widowed/never	1.31 (1.04-1.66)	0.022*
From married to divorced/widowed	0.86 (0.64-1.16)	0.321
From divorced/widowed/never to married	1.17 (0.66-2.06)	0.591
**Change in economic status**		
Keep favorable	[1]	
Keep unfavorable	1.50 (1.07-2.11)	0.018*
From favorable to unfavorable	1.43 (1.14-1.79)	0.002**
From unfavorable to favorable	1.00 (0.998-1.002)	0.762
**Change in living pattern**		
Keep Living with family members	[1]	
Keep living alone or at nursing home	0.83 (0.63-1.09)	0.174
From living with family members to living alone or at nursing home	0.85 (0.63-1.14)	0.274
From living alone or at nursing home to living with family members	1.07 (0.84-1.35)	0.591
**Change in ADL**	1.06 (1.03-1.09)	<0.001***
**Change in IADL**	1.06 (1.05-1.08)	<0.001***
**Change in BMI**	1.00 (0.998-1.002)	0.762
**Change in smoking status**		
Keep smoking	[1]	
Keep not smoking	1.14 (0.86-1.52)	0.361
Quit smoking	1.19 (0.81-1.75)	0.385
Start smoking	1.12 (0.72-1.76)	0.613
**Change in drinking alcohol status**		
Keep drinking	[1]	
Keep not drinking	1.09 (0.79-1.50)	0.599
Quit drinking	1.07 (0.73-1.58)	0.719
Start drinking	1.10 (0.74-1.66)	0.634
**Change in doing exercise status**		
Keep exercising	[1]	
Keep not exercising	1.62 (1.22-2.15)	0.001**
Stop exercising	1.45 (1.07-1.98)	0.017*

Start exercising	1.79 (1.30-2.46)	<0.001***
**Change in hypertension**		
Keep having	[1]	
Keep not having	1.16 (0.91-1.48)	0.229
Recover	1.35 (0.98-1.85)	0.063
Start having	0.99 (0.75-1.32)	0.954
**Change in diabetes**		
Keep having	[1]	
Keep not having	0.85 (0.43-1.70)	0.654
Recover	0.74 (0.20-2.77)	0.653
Start having	0.51 (0.21-1.21)	0.125
**Change in heart disease**		
Keep having	[1]	
Keep not having	1.13 (0.75-1.69)	0.561
Recover	1.18 (0.69-2.02)	0.544
Start having	0.93 (0.58-1.49)	0.764
**Change in stroke or CVD**		
Keep having	[1]	
Keep not having	0.56 (0.32-0.99)	0.045*
Recover	0.55 (0.27-1.13)	0.105
Start having	0.63 (0.34-1.15)	0.132
**Change in cataract**		
Keep having	[1]	
Keep not having	1.07 (0.64-1.79)	0.791
Recover	1.37 (0.77-2.44)	0.287
Start having	1.04 (0.60-1.81)	0.889
**Change in digestive system diseases**		
Keep having	[1]	
Keep not having	0.72 (0.34-1.53)	0.395
Recover	0.56 (0.24-1.34)	0.192
Start having	0.61 (0.26-1.45)	0.262
**Change in arthritis**		
Keep having	[1]	
Keep not having	0.86 (0.58-1.27)	0.456
Recover	1.01 (0.67-1.54)	0.949
Start having	1.22 (0.78-1.92)	0.381
**Change in Parkinson's disease**		
Keep having	[1]	
Keep not having	0.17 (0.02-1.32)	0.090
Recover	0.28 (0.03-2.96)	0.290
Start having	0.13 (0.01-1.38)	0.091
**Change in overall protein intake**	0.95 (0.92-0.99)	0.008**

*p<0.05 **p<0.01 ***p<0.001

Table S2. The association between the change in different types of protein intake and cognitive impairment

	HR (95% CI)	P value
**Animal-based protein**	0.98 (0.96-0.997)	0.021*
Meats	0.96 (0.91-1.003)	0.066
Fish	0.95 (0.91-0.999)	0.045*
Eggs	0.94 (0.90-0.99)	0.014*
Milk* and *dairy products	1.00 (0.96-1.04)	0.945
**Plant-based protein**	0.96 (0.93-0.99)	0.007**
Bean products	0.93 (0.89-0.97)	0.002**
Nuts	0.98 (0.93-1.03)	0.433

*p<0.05 **p<0.01

Adjusted for baseline age (continuous), baseline gender, baseline residence, baseline years of schooling, change in marital status, change in economic status, change in living pattern, change in tobacco smoking, change in alcohol drinking, change in regular exercise, change in ADL, change in IADL, change in BMI, change in chronic disease (hypertension, diabetes, heart disease, stroke or CVD, cataract, digestive system disease, arthritis, Parkinson's disease).

Table S3**.** The association between the change in different types of protein intake between baseline and the first follow-up and cognitive impairment in the second follow-up.

	OR (95% CI)	P value
**Total protein**	0.99 (0.97-1.01)	0.373
**Animal-based protein**	0.99 (0.96-1.02)	0.498
Meats	0.92 (0.85-1.01)	0.070
Fish	0.92 (0.84-0.99)	0.034*
Eggs	0.97 (0.89-1.05)	0.396
Milk* and *dairy products	1.09 (1.02-1.17)	0.013*
**Plant-based protein**	0.98 (0.93-1.03)	0.393
Bean products	0.95 (0.88-1.02)	0.165
Nuts	1.01 (0.93-0.10)	0.841

*p<0.05

Adjusted for age (continuous), gender, residence, years of schooling, marital status, economic status, living pattern, tobacco smoking, alcohol drinking, regular exercise, ADL, IADL, BMI, chronic disease (hypertension, diabetes, heart disease, stroke or CVD, cataract, digestive system disease, arthritis, Parkinson's disease)
